# Effects of global transcription factor NtcA on photosynthetic production of ethylene in recombinant *Synechocystis* sp. PCC 6803

**DOI:** 10.1186/s13068-017-0832-y

**Published:** 2017-06-06

**Authors:** Huilin Mo, Xiaoman Xie, Tao Zhu, Xuefeng Lu

**Affiliations:** 1 Key Laboratory of Biofuels, Shandong Provincial Key Laboratory of Synthetic Biology, Qingdao Institute of Bioenergy and Bioprocess Technology, Chinese Academy of Sciences, No. 189 Songling Road, Qingdao, 266101 China; 2 University of Chinese Academy of Sciences, Beijing, 100049 China

**Keywords:** *Synechocystis* sp. PCC 6803, Ethylene, NtcA, TCA cycle, Glycogen

## Abstract

**Background:**

Cyanobacteria are considered potential photosynthetic microbial cell factories for biofuel and biochemical production. Ethylene, one of the most important organic chemicals, has been successfully synthesized in cyanobacteria by introducing an exogenous ethylene-forming enzyme (Efe). However, it remains challenging to significantly improve the biosynthetic efficiency of cyanobacterial ethylene. Genetic modification of transcription factors is a powerful strategy for reprogramming cellular metabolism toward target products. In cyanobacteria, nitrogen control A (NtcA), an important global transcription regulator of primary carbon/nitrogen metabolism, is expected to play a crucial role in ethylene biosynthesis.

**Results:**

The partial deletion of *ntcA* (MH021) enhanced ethylene production by 23%, while *ntcA* overexpression (MH023) in a single-copy *efe* recombinant *Synechocystis* (XX76) reduced ethylene production by 26%. Compared to XX76, the Efe protein content increased 1.5-fold in MH021. This result may be due to the release of the negative regulation of NtcA on promoter P_*cpcB*_, which controls *efe* expression. Glycogen content showed a 23% reduction in MH021, and the ratio of intracellular succinate to 2-oxoglutarate (2-OG) increased 4.8-fold. In a four-copy *efe* recombinant strain with partially deleted *ntcA* and a modified tricarboxylic acid (TCA) cycle (MH043), a peak specific ethylene production rate of 2463 ± 219 μL L^−1^ h^−1^ OD_730_^−1^ was achieved, which is higher than previously reported.

**Conclusions:**

The effects of global transcription factor NtcA on ethylene synthesis in genetically engineered *Synechocystis* sp. PCC 6803 were evaluated, and the partial deletion of *ntcA* enhanced ethylene production in both single-copy and multi-copy *efe* recombinant *Synechocystis* strains. Increased Efe expression, accelerated TCA cycling, and redirected carbon flux from glycogen probably account for this improvement. The results show great potential for improving ethylene synthetic efficiency in cyanobacteria by modulating global regulation factors.

**Electronic supplementary material:**

The online version of this article (doi:10.1186/s13068-017-0832-y) contains supplementary material, which is available to authorized users.

## Background

An increased consumption of fossil resources has accelerated the development of alternative routes for producing renewable fuels and chemicals. Photoautotrophic cyanobacteria are promising solar biocatalysts for the production of various target products due to their genetic tractability, fast growth, and high photosynthetic efficiency [1, 2]. Ethylene, a widely used raw material in the chemical industry and in consumer markets, has already been synthesized in model strains *Synechococcus elongatus* PCC 7942 (hereafter called *S. elongatus* PCC 7942) [3] and *Synechocystis* sp. PCC 6803 (hereafter called *S*. PCC 6803) [4–8] by introducing an extrinsic ethylene-forming enzyme (Efe) pathway [9, 10].

Many efforts have been devoted to constructing stable and efficient ethylene-producing strains in recent years, including (1) optimizing host-preferred codons for *efe* expression [5, 7], (2) screening more efficient promoters [5, 6], (3) increasing copy numbers of *efe* [5, 7], (4) modifying ribosome binding sites upstream of *efe* [8], and (5) modifying related metabolic pathways [5]. In addition to genetic manipulations, a cultivation process for ethylene production was also optimized, considering factors such as light intensity, medium components, and CO_2_ supply [5, 7]. A volumetric ethylene production rate of 9.7 mL L^−1^ h^−1^ was achieved in our previous study by introducing three copies of *efe* in *S*. PCC 6803 with blockage of the 2-oxoglutarate decarboxylase (OGDC) and γ-aminobutyrate (GABA) shunts of the tricarboxylic acid (TCA) cycle and expression of 2-OG permease from *E*. *coli*; this is the highest cyanobacterial ethylene productivity to our knowledge [5]. Recently, it was found that *S*. PCC 6803 harbors a complete TCA cycle with a low flux (13% of total fixed carbon) [8, 11] and that an exogenous Efe pathway can turn the bifurcated architecture into a cyclic pathway with an enhanced carbon flux (37% of total fixed carbon) [8]. With an in-depth understanding of the plasticity of cyanobacterial metabolism, a global rebalance of cellular carbon and nitrogen metabolism is important to develop more powerful ethylene photosynthetic cell factories. Global transcription machinery engineering (gTME) [12] has already been successfully applied to regulate primary metabolism toward target products in both prokaryotic and eukaryotic systems [13]. An improvement in cyanobacterial production of polyhydroxybutyrate (PHB, product of *phaAB* operon) [14] and hydrogen [15] has been reported by overexpression of *rre37* (encoding response regulator 37) or *sigE* (encoding RNA polymerase sigma factor E), suggesting the huge potential of using the gTME method to enhance target metabolite production in cyanobacteria.

**Fig. 1:**
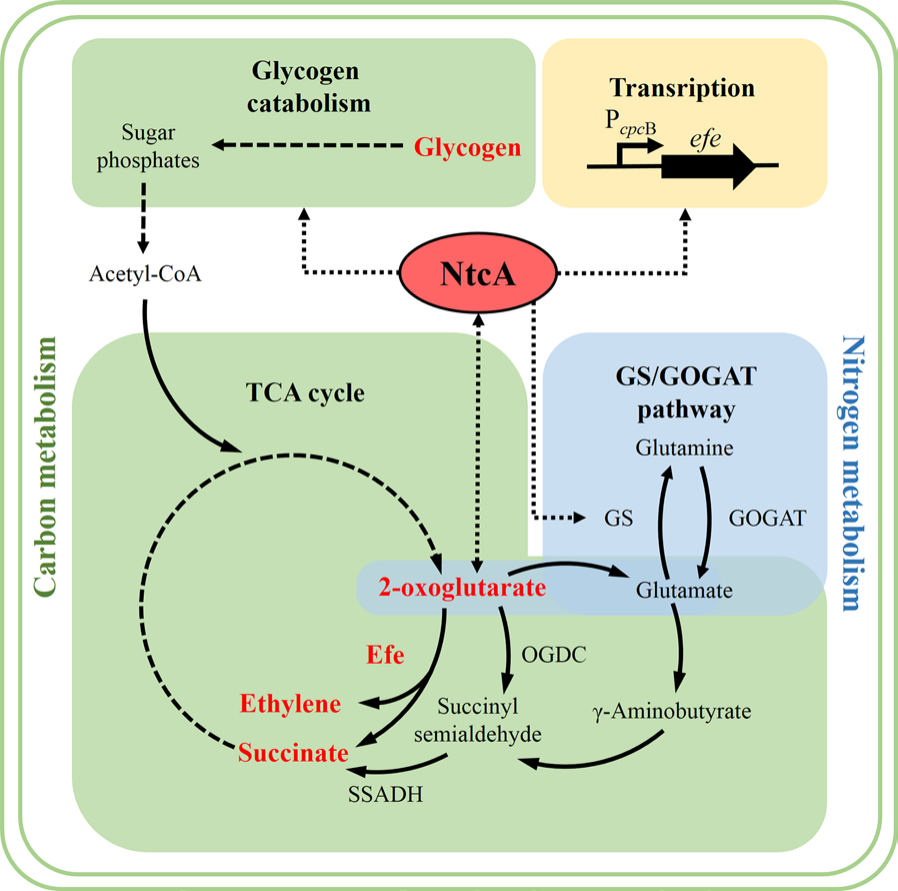
Schema illustrating the involvement of NtcA in carbon and nitrogen metabolism and ethylene production. GS, glutamine synthetase; GOGAT, glutamine oxoglutarate amidotransferase (glutamate synthase); OGDC, 2-oxoglutarate decarboxylase; SSADH, succinic semialdehyde dehydrogenase

As a key precursor of ethylene biosynthesis, 2-OG is one of the most important indicators of carbon/nitrogen metabolic balance. The 2-OG pool is regulated by global transcription factor nitrogen control A (NtcA), which implies that NtcA should play a crucial role in ethylene production in cyanobacteria. The glutamine synthetase (GS, encoded by *glnA*)/glutamine oxoglutarate aminotransferase (GOGAT, encoded by *glsF* or *gltB*/*gltD*) [16, 17] cycle is a pathway that represents a connecting step between carbon and nitrogen metabolism in *S*. PCC 6803 (Fig. 1). GS is a key enzyme in nitrogen assimilation, and it is regulated in multiple ways by NtcA, e.g., NtcA can positively modulate the transcription of *glnA* [18], negatively regulate the transcription of *gifA* and *gifB* (encoding GS inactivating factors IF7 and IF17, respectively) [18, 19], and positively modulate the transcription of nitrogen stress-induced RNA 4 (NsiR4, negative regulator of IF7) [20]. In addition to genes related to nitrogen metabolism, NtcA regulates genes in a variety of other cellular processes (such as carbon metabolism and photosynthesis) as well as several sigma factors [20, 21]. Although it was reported that *ntcA* can only be partially deleted in *S*. PCC 6803 [19, 22], a non-completely segregated mutant exhibited abolished activations of *ntcA* on positive regulons (e.g., *glnA*, *glnB*, and *glnN* promoters) [22] and repressions of *ntcA* on negative regulons (e.g., *gifA* and *gifB* promoters) [19]. On the other hand, overexpression of *ntcA* leads to wide alterations in primary metabolism and a close to 90% loss of the intracellular 2-OG pool [23]. In addition, NtcA directly acts on sugar catabolism, which is indispensable to metabolism in cyanobacteria, by activating the transcription of *sigE* [24] and *rre37* [25]. These results revealed that genetic modification of *ntcA* severely disturbed cellular metabolism in *S*. PCC 6803.

In this study, *ntcA* was chosen as a genetic engineering target to evaluate its effects on ethylene production in *S*. PCC 6803. We constructed *ntcA* partial deletion mutants and *ntcA* overexpression mutants using *S*. PCC 6803 wild-type (WT) and ethylene-producing *Synechocystis* recombinants as the parent strains. In addition, we analyzed the Efe protein level, glycogen content, levels of the substrate and the accompanying product of the Efe-catalyzed reaction (2-OG and succinate of TCA cycle, respectively), and ethylene productivity in these strains.

## Methods

### Chemicals and reagents

The kits used for molecular cloning were from TransGen (China) or Takara (Japan). *Taq* DNA polymerase and restriction enzymes were obtained from Fermentas (Canada) or Takara (Japan). The antibodies and chemicals used for western blotting were from Sangon Biotech (China). Pure ethylene gas standard was purchased from Heli Gas Co., Ltd (China). The α-ketoglutarate assay kit was ordered from Sigma-Aldrich (USA). Unless otherwise specified, all other chemical reagents were purchased from Sigma-Aldrich (USA).

### Strain and plasmid construction

All plasmids, strains, and primers used in this study are listed in Table 1, and the details are described in this section. Clones of the PCR products were confirmed by sequencing.

**Table 1:** Plasmids, strains, and primers used in this study

Plasmids, strains, and primers	Derivation and/or relevant characteristics	References or source
*Plasmids*		
pMD19-Ts	Ap^r^, cloning vector	Takara
pFL-XS	Ap^r^Cm^r^, a BioBrick “T” vector used for functional block assembling	[5]
pHM001	Ap^r^, pMD19-Ts derivative containing the *sll1423* ORF with flanking regions	This study
pHM002	Ap^r^Km^r^, pMD19-Ts derivative used to knock out *sll1423* (*ntcA*) by insertional inactivation with kanamycin resistance cassette	This study
pHM003	Ap^r^Km^r^, pMD19-Ts derivative used to delete *sll1423* (*ntcA*) with kanamycin resistance cassette	This study
pHM004	Ap^r^, pFL-XS derivative containing the *sll1423* ORF	This study
pHM005	Ap^r^Gm^r^, pFL-XS derivative containing Gm^r^-P_*cpcB*_-*ntcA* expression cassette	This study
pHM006	Ap^r^Gm^r^, pFL-XS derivative used to express Gm^r^-P_*cpcB*_-*ntcA* expression cassette at *phaAB* locus	This study
pHM008	Ap^r^Gm^r^, pMD19-Ts derivative used to inactivate *sll1423* with the gentamycin resistance cassette	This study
pHM009	Ap^r^Gm^r^, pFL-XS derivative containing Gm^r^-P_*cpcB*_-*efe* expression cassette	This study
pHM010	Ap^r^Gm^r^, pFL-XS derivative used to express Gm^r^-P_*cpcB*_-*efe* expression cassette at *sll1423* locus	This study
pXX55	Ap^r^, pFL-XS derivative containing P_*cpcB*_-*efe* expression cassette	[5]
pXX58	Ap^r^Gm^r^, pFL-XS derivative containing the Gm^r^ cassette *aacC1*	[5]
pXX62	Ap^r^Gm^r^, pFL-XS derivative containing Gm^r^-P_*cpcB*_ cassette	[5]
pKC104	Ap^r^, pMD18-Ts derivative containing upstream and downstream fragments of *phaAB* operon	[26]
pRL446	Ap^r^Km^r^, plasmid containing the Km^r^ cassette	Prof. X. Xu
*Synechocystis strains*		
*S*. PCC 6803	*Synechocystis* sp. PCC 6803 wild-type	Prof. X. Xu
XX76	*slr0168*::Sp^r^-P_*cpcB*_-*efe*	[5]
MH013	*sll1423*::Km^r^ (Insertional inactivation)	This study
MH015	*sll1423*::Km^r^ (Deletion)	This study
MH017	*phaAB*::Gm^r^-P_*cpcB*_-*ntcA*	This study
MH021	*slr0168*::Sp^r^-P_*cpcB*_-*efe*/*sll1423*::Km^r^	This study
MH023	*slr0168*::Sp^r^-P_*cpcB*_-*efe*/*phaAB*::Gm^r^-P_*cpcB*_-*ntcA*	This study
XX109	*slr0168*::Sp^r^-P_*cpcB*_-efe/*sll1981*::Km^r^-P_*cpcB*_-*efe*/s*lr0370*::Cm^r^-P_*cpcB*_-*efe*	[5]
MH039	*slr0168*::Sp^r^-P_*cpcB*_-efe/*sll1981*::Km^r^-P_*cpcB*_-*efe*/s*lr0370*::Cm^r^-P_*cpcB*_-*efe*/*sll1423*::Gm^r^	This study
MH043	*slr0168*::Sp^r^-P_*cpcB*_-efe/*sll1981*::Km^r^-P_*cpcB*_-*efe*/s*lr0370*::Cm^r^-P_*cpcB*_-*efe*/*sll1423*::Gm^r^-P_*cpcB*_-*efe*	This study
*Primers (5′→3′)* ^a^		
Detection primers for *ntcA* site (*slr1423*) in single-copy *efe* recombinants		
ntcA-d1	GTTACTCAGCACAACGGGGTC	
ntcA-d2	TTGCAGCCCTTCGCCAGCTGGCACGTTCACGGTAATGGGG	*Pvu*II
kan-1	CCCCATTACCGTGAACGTGCCAGCTGGCGAAGGGCTGCAA	*Pvu*II
kan-2	GCACTGGTCATAGAGGGTGGCAGCTGGCACGACAGGTTTC	*Pvu*II
ntcA-d3	GAAACCTGTCGTGCCAGCTGCCACCCTCTATGACCAGTGC	*Pvu*II
ntcA-d4	TAACTGACCCCGCAGAATGGC	
ntcA-x1	ATGGATCAGTCCCTAACCC	
ntcA-x2	TTAGGTAAACTGTTGACTGAGAGC	
phaAB-3	GCCTTGGGCTAAGTTATTGAGCG	
phaAB-4	TAGGATTCTTGCACAGTACCGC	
*Quantitative real-time PCR*		
rnpB-f1	TGAGGACAGTGCCACAGAA	
rnpB-f2	AATTCCTCAAGCGGTTCCAC	
rrn16Sa-f1	CCAACATCTCACGACACGA	
rrn16Sa-f2	ACTAGGCGTGGCTTGTATCG	
ntcA-f1	CGGCGGAACGGGTTTATT	
ntcA-f2	CAATGACAGGTCGGGATGC	
*Detection primers for ntcA site (slr1423) in multi-copy efe recombinants*		
ntcA-5	GGCAGTGTGGAGCGCATGTAAT	
ntcA-6	AGTAATCACCGTCAACAATACCGC	
slr0168-1	ACCTCTCCACGCTGAATTAG	[5]
slr0168-2	TTCCAGGCCACATTGTTGTC	[5]
sll1981-1	GGGCTTCGTTAGGTTGTGTGGC	[5]
sll1981-2	CCGCATGGCCGTTTCCAACTCC	[5]
slr0370-1	GCCGAGGAATACTTAGCCGATG	[5]
slr0370-2	CTGCCCTATGAACCGAATATGG	[5]

Ap, ampicillin; Km, kanamycin; Sp, spectinomycin; Cm, chloramphenicol; Gm, gentamycin

^a^Restriction enzyme sites added in the primers were underlined and listed

#### Plasmids used to knock out *ntcA* (*sll1423*) by insertional inactivation

The open reading frame (ORF) of *ntcA* and its flanking regions were amplified by PCR from *S*. PCC 6803 genomic DNA using primers ntcA-d1/d4. The amplified PCR fragment was cloned into pMD19-T simple (Takara), resulting in pHM001. Plasmid pHM001 was digested completely with *Bam*HI and *Bgl*II to remove the major coding regions of *ntcA* (465 bp) and was blunted with T4 DNA polymerase. Then, the plasmid was ligated with the kanamycin resistance (Km^r^) cassette excised from pRL446 with *Pvu*II, resulting in pHM002. Plasmid pHM002, which harbors the *ntcA* upstream flanking region (*ntcA*U) with 125 bp of the 5′ end of *ntcA*, Km^r^ cassette, and *ntcA* downstream-flanking region (*ntcA*D) with 88 bp of the 3′ end of *ntcA*, was used to knock out *ntcA* by insertional inactivation.

#### Plasmids used to knock out *ntcA* (*sll1423*) by deletion

The flanking regions of *ntcA* (*ntcA*U 883 bp and *ntcA*D 905 bp) were amplified by PCR from *S*. PCC 6803 genomic DNA using primers ntcA-d1/d2 and ntcA-d3/d4, respectively. The Km^r^ cassette was amplified by PCR from pRL446 using primers kan-1/2. The *ntcA*U-Km^r^-*ntcA*D cassette was generated by fusion PCR using primers ntcA-d1/d4 and was inserted into pMD19-T simple, resulting in pHM003. Plasmid pHM003, which harbors the *ntcA* upstream flanking region, Km^r^ cassette and *ntcA* downstream-flanking region, was used to knock out *ntcA* by deletion.

#### Plasmids used to overexpress *ntcA* (*sll1423*) at the *phaAB* loci

The coding region of *ntcA* was amplified by PCR from *S*. PCC 6803 genomic DNA using primers ntcA-x1/x2 and was inserted into the *Xcm* I-digested pFL-XS BioBrick “T” vector that we previously constructed [5], resulting in pHM004. The DNA fragment containing the promoter region of the *cpcB* gene (gentamycin resistance (Gm^r^)-P_*cpcB*_ cassette) was excised from pXX62 with *Eco*RI and *Spe*I and was inserted into the *Eco*RI and *Xba*I sites of pHM004 to drive the expression of *ntcA*, resulting in pHM005. The Gm^r^-P_*cpcB*_-*ntcA* expression cassette was excised from pHM005 with *Eco*RI and *Hind*III, blunted with T4 DNA polymerase, and inserted into the *Nco*I site (blunted) of pKC104 [26] with the 5′ and 3′ end flanking regions of the *phaAB* loci, resulting in pHM006. Plasmid pHM006 was used to simultaneously overexpress *ntcA* and block polyhydroxybutyrate biosynthesis.

#### Plasmids used to knock out *ntcA* and express *efe* at the *ntcA* locus in multi-copy *efe* recombinants

The Gm^r^ cassette was excised from pXX58 with *Eco*RI and *Hind*III, blunted with T4 DNA polymerase, and inserted into *Eco*RI-digested and blunted *ntcA* deletion plasmid pHM003, resulting in pHM008, which was used to disrupt *ntcA* in strain XX109 (three-copy *efe*). The P_*cpcB*_-*efe* cassette was excised from pXX55 with *Eco*RI and *Xba*I and was inserted into the *Eco*RI and *Spe*I sites of pXX58, resulting in pHM009. The Gm^r^-P_*cpcB*_-*efe* expression cassette was excised from pHM009 with *Eco*RI and *Hind*III, blunted with T4 DNA polymerase, and inserted into *Eco*RI-digested, T4 DNA polymerase-blunted *ntcA* deletion plasmid pHM003, resulting in pHM010. Plasmid pHM010 was used to introduce another *efe* copy at the *ntcA* site.

### Generation of cyanobacterial transformants

Transformation of WT and mutant *S*. PCC 6803 was performed according to the established procedures [27]. Plasmids pHM002 and pHM003 were transformed to *S.* PCC 6803 to generate the *ntcA* disruption strains MH013 and MH015, respectively, and pHM006 was transformed to generate the *ntcA* overexpression strain MH017. Plasmids pHM003 and pHM006 were transformed to the one-copy *efe* recombinant strain XX76 to generate MH021 and MH023, respectively. Plasmid pHM008 was transformed to the three-copy *efe* recombinant strain XX109 to generate MH039. Plasmid pHM010 was transformed to XX109 to generate the four-copy *efe Synechocystis* recombinant MH043. All the *Synechocystis* mutants constructed here are listed in Table 1, and their genotypes were confirmed by PCR using the corresponding primers listed in Table 1.

### Cultivation of *E. coli* and *Synechocystis*

*Escherichia coli* was grown using standard procedures in Luria–Bertani (LB) medium at 37 °C. The *Synechocystis* strains were grown in standard BG11 medium that was bubbled with air at 30 °C under approximately 50–100 μmol photons m^−2^ s^−1^ illumination of white light.

For BG11 solid medium, the culture was supplemented with 1.4% agar, 8 mM TES–NaOH (pH 8.2), and 0.3% Na_2_S_2_O_3_. The culture was supplemented with appropriate antibiotics when necessary (kanamycin, 25 μg mL^−1^; spectinomycin, 10 μg mL^−1^; chloramphenicol, 10 μg mL^−1^; gentamycin, 10 μg mL^−1^; when two or more antibiotics were used, the concentrations of the antibiotics were reduced by half). Growth and cell densities were measured at OD_730_ with a T6 UV/VIS spectrophotometer (Persee, China).

### Quantification PCR (qPCR) of *ntcA* copy numbers

The genomic DNA was purified with a Genomic DNA Purification Kit (Sangon Biotech, China). Amplification was performed using SYBR^®^ Premix Ex Taq™ II (Takara) with gene-specific primers (Table 1) and genomic DNA of triplicate technical replicates from duplicate biological cultures. The *rnpB* (encoding the RNA subunit of ribonuclease P) and *rrn16Sa* (encoding the 16S ribosomal RNA) genes were used as reference genes. The primers used in qPCR are listed in Table 1: rnpB-f1/f2, with a product size of 246 bp; rrn16Sa-f1/f2, with a product size of 266 bp; and ntcA-f1/f2, with a product size of 242 bp. The final PCR mixture contained primers at a final concentration of 625 nM in a total volume of 20 μL. The two-step cycling conditions were 10 min at 95 °C and 45 cycles at 95 °C for 20 s and 60 °C for 1 min. The relative genomic DNA copy numbers were determined with LightCycler 480 software according to the 2^−ΔΔCT^ method [28]. To correctly calculate the relative genomic DNA copy number, PCR efficiency was determined by a dilution series of the genomic DNA.

### Ethylene production assay

Ethylene production was determined as described in our previous report [5]. The quantitative analysis of ethylene production was performed with the help of an ethylene calibration curve (Additional file 1: Figure S1).

### Spectrophotometric determination of pigment contents

The *Synechocystis* strains were inoculated at OD_730_ ~ 0.1 and were grown photoautotrophically. An appropriate aliquot of culture was centrifuged at the indicated times (the 2nd, 3rd, and 4th days, respectively) and was resuspended in fresh BG11 to OD_730_ ~ 0.5. Then the whole-cell absorbance spectra (550–750 nm) were recorded on a Beckman Coulter DU-800 UV/VIS spectrophotometer. The pigment contents were calculated based on the absorbance maxima as follows: 630 nm for phycocyanin (PC) and 680 nm for chlorophyll a (Chl a).

### Protein extraction and western blotting analysis

Fifty milliliters of cell culture grown for 4 days (OD_730_ ~ 1.0) was centrifuged, resuspended in 500 μL of 20 mM phosphate buffer (Na_2_HPO_4_ and NaH_2_PO_4_, pH 7.4), and disrupted with glass beads (0.25–0.30 mm) by vigorous vortexing (45 min in cycles of mixing for 40 s and cooling for 20 s). The supernatant was collected after centrifugation for western blotting analysis, which was performed as described previously [5].

### Organic acid measurement

#### Determination of intracellular succinate

Intracellular succinate was extracted as previously described [8], with minor revisions. Ten milliliters of cell culture grown for 4 days (OD_730_ ~ 1.0) was filtered onto a sterile nitrocellulose membrane (0.45 μm, Jinteng, China). Then the pellets were scraped from the filter membrane into cold (−80 °C) methanol. After the pellets were thoroughly mixed with chloroform and centrifugation, the supernatant was collected and evaporated by vacuum centrifugation. Next, the extract was re-dissolved in 200 μL of 50% methanol:water (V/V) and was detected by Agilent 1200 High-Performance Liquid Chromatography (USA) using an Aminex HPX-87-H column (300 × 7.8 mm) detected at 210 nm. The column was operated at 60 °C with a mobile phase of 5 mM H_2_SO_4_ at a flow rate of 0.5 mL min^−1^.

#### Determination of intracellular 2-OG

Intracellular 2-OG was extracted as previously described [18], with minor revisions. Fifty milliliters of cell culture grown for 4 days (OD_730_ ~ 1.0) was harvested by centrifuging at 10,000*g* for 5 min at 4 °C. The pellets were resuspended in 500 μL of cold 0.3 M HClO_4_ and were incubated for 15 min on ice. The resulting lysates were centrifuged at 15,000*g* for 15 min at 4 °C. Then the supernatant was neutralized by the addition of 37.5 μL of cold 2 M K_2_CO_3_. The KClO_4_ precipitate was removed by centrifugation, and the supernatant containing 2-OG was stored at −80 °C for analysis. The intracellular 2-OG concentration was determined using the α-ketoglutarate assay kit (Sigma-Aldrich, USA).

### Glycogen measurement

Glycogen content was measured as described previously [29], with minor modification. To isolate glycogen, a certain amount of cells, equivalent to 10 OD_730_ units of *Synechocystis* culture, was collected and rinsed twice with 1 mL of deionized water. After centrifugation, the cells were suspended in 30% (W/V) KOH and were incubated at 95 °C for 2 h. Glycogen was then precipitated by the addition of ice-cold ethanol to a final concentration of 70–75% (V/V) and was subsequently incubated at −20 °C overnight. After centrifugation, the glycogen pellet was washed twice with 70 and 100% (V/V) ethanol and was dried by vacuum centrifugation at 65 °C. The isolated glycogen was resuspended in 100 mM sodium acetate (pH 4.5) and was digested to glucose by glucosidase at 65 °C for 2 h. Soluble glucose from the digests was determined using an SBA-40c biosensor analyzer (Shandong Academy of Sciences, China) equipped with a glucose oxidase-immobilized membrane. To determine dry cell weight (DCW), cells were filtered through sterile nitrocellulose membranes (0.45 μm, Jinteng, China), washed twice with 1 mL of deionized water, and dried at 105 °C for 24 h. Then the content of glycogen was calculated based on the weight of the dried cells equivalent to those were used for glycogen determination.

## Results

### Inactivation and overexpression of *ntcA* in *S*. PCC 6803 and ethylene-producing recombinants

**Fig. 2:**
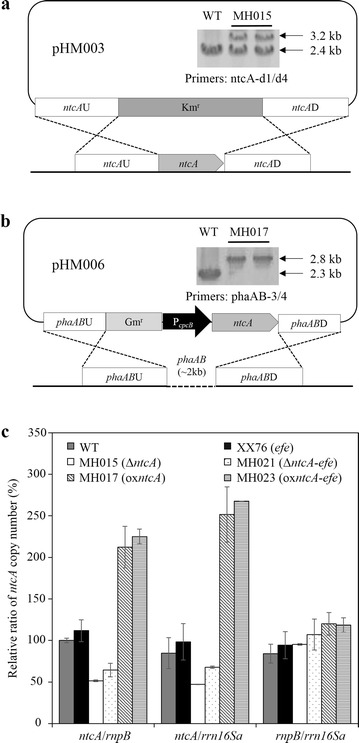
Inactivation and overexpression of *ntcA* in *S*. PCC 6803 WT and ethylene-producing recombinant. **a** Schematic representation of the deletion of *ntcA*. The *open reading frame* (ORF) of *ntcA* (*sll1423*) was replaced by a kanamycin resistance (Km^r^) cassette through homologous recombination with plasmid pHM003. DNA fragments were amplified by PCR and were analyzed by agarose gel electrophoresis, showing the partial segregation of *ntcA* in the mutant strains. PCR products from *S*. PCC 6803 were generated using the indicated primer pairs. Primer sequences are listed in Table 1. *Lanes* were loaded with PCR products that were generated with genomic DNA from the indicated strain as a template. The sizes of the PCR products are indicated on the *right*. **b** Schematic representation of the overexpression of *ntcA*. The Gm^r^-P_*cpcB*_-*ntcA* expression cassette was inserted into the *phaAB* loci through homologous recombination with plasmid pHM006. DNA fragments were amplified by PCR and were analyzed by agarose gel electrophoresis, which showed the complete replacement of *phaAB* by the Gm^r^-P_*cpcB*_-*ntcA* expression cassette. PCR products from *S*. PCC 6803 were generated using the indicated primer pairs. Primer sequences are listed in Table 1. *Lanes* were loaded with the PCR products that were generated with genomic DNA from the indicated strains as templates. The sizes of the PCR products are indicated on the right. **c** Quantitative PCR results of *ntcA* deletion and overexpression mutants. The copy numbers of *ntcA* were measured through qPCR. The reference genes for *S*. PCC 6803 were *rnpB* and the 16S rRNA gene. The relative ratios of gene copy numbers of *ntcA* were quantified in the WT strain, ethylene producer XX76, and *ntcA* mutant strains (MH015, MH021, MH017, and MH023). Data represent the means ± standard deviations from three independent experiments

With the aim of evaluating the effect of NtcA on ethylene production, we first inactivated the transcription factor in *S*. PCC 6803. Two strategies, insertional inactivation and deletion, were employed, and the resultant mutants were denoted as MH013 (Additional file 1: Figure S2) and MH015 (Fig. 2a), respectively. Unlike *ntcA* of *S. elongatus* PCC 7942 and *Anabaena* sp. PCC 7120, which can be completely inactivated (using ammonium instead of NaNO_3_ in BG11 medium, hereafter referred to as BG11_A_) [30, 31], we found that both MH013 (*ntcA* insertional inactivation) (Additional file 1: Figure S2) and MH015 (*ntcA* deletion) (Fig. 2a) were only partially inactivated. These findings are consistent with previous reports that NtcA is essential for *S*. PCC 6803 and cannot be fully knocked out when cultured in BG11 or BG11_A_ medium [19, 22]. Next, we constructed MH021 (Additional file 1: Figure S3a, b) by deleting *ntcA* in XX76 (single-copy *efe*). For segregation-level analysis, qPCR was performed to measure the relative gene copy number of *ntcA* in MH015 (*ntcA* deletion) and MH021 (*ntcA* deletion in single-copy *efe*). Our results indicated that MH015 and MH021 exhibited approximately 51 and 34% loss of *nctA* WT copies relative to their starting strains, *S*. PCC 6803 and XX76, respectively (Fig. 2c).

We also overexpressed *ntcA* under the control of the P_*cpcB*_ promoter on the *phaAB* loci in *S*. PCC 6803 and XX76, denoted as MH017 (*ntcA* overexpression) and MH023 (*ntcA* overexpression in single-copy *efe*), respectively (Fig. 2b, Additional file 1: Figure S3a, b). Successful integration of the Gm^r^-P_*cpcB*_-*ntcA* cassette was achieved, and qPCR revealed that the relative gene copy number of *ntcA* increased by more than twofold in both MH017 and MH023 compared to their respective starting strains (Fig. 2c).

Moreover, we found that the deletion and overexpression mutants were both genetically stable with a similar segregation level after several rounds of subculture.

### Ethylene production was enhanced by *ntcA* inactivation and was reduced by *ntcA* overexpression

**Fig. 3:**
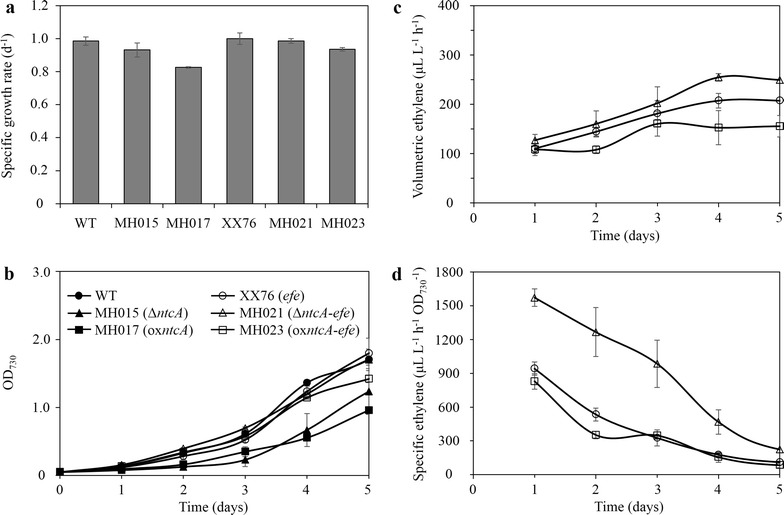
Effect of *ntcA* on cell growth and ethylene productivity. **a** Specific growth rate of *S*. PCC 6803 WT, XX76 (single-copy *efe*), MH015 (*ntcA* deletion), MH017 (*ntcA* overexpression), MH021 (*ntcA* deletion in single-copy *efe*), and MH023 (*ntcA* overexpression in single-copy *efe*). **b** Growth curves. **c** Volumetric ethylene production. **d** Specific ethylene productivity. Data represent the means ± standard deviations from three independent experiments

After the generation of *ntcA* deletion and overexpression mutants, specific growth rates were evaluated in standard BG11 medium. As shown in Fig. 3a, the two starting strains, *S*. PCC 6803 and XX76 (single-copy *efe*), showed similar specific growth rates. However, *ntcA* partial deletion mutant MH015 and *ntcA* overexpression strain MH017 both showed restrained growth (Fig. 3b). Intriguingly, the specific growth rates of the ethylene producer (MH021) with *ntcA* partial deletion and the *ntcA* overexpression recombinant (MH023) recovered in comparison to the growth rates of MH015 and MH017, respectively. Strain MH021 even exhibited a similar specific growth rate to those of *S*. PCC 6803 and XX76 (Fig. 3a).

Ethylene production was also monitored, and the results indicated that all ethylene producers reached their peak production in terms of volumetric ethylene production in the first 4 days (Fig. 3c). Ethylene production in the *ntcA* overexpression recombinant (MH023) was maximally reduced by 26% compared to that of the baseline strain XX76 (Fig. 3c). In contrast, volumetric ethylene production in the *ntcA* partial deletion mutant (MH021) was maximally increased by 23% compared to that of XX76 (Fig. 3c). Regarding specific ethylene production, MH021 reached a peak value of 1572 ± 77 μL L^−1^ h^−1^ OD_730_^−1^, which was 1.7-fold higher than that of XX76. However, MH023 (*ntcA* overexpression in single-copy *efe*) generally showed slightly reduced or unchanged specific ethylene production, despite its 7% lower specific growth rate (Fig. 3a, d). Thus, our results indicate that the deficiency of *ntcA* is an effective strategy for enhancing ethylene production in *S*. PCC 6803.

### Deficiency of *ntcA* enhanced the expression of the P_*cpcB*_-controlled exogenous *efe* gene

**Fig. 4:**
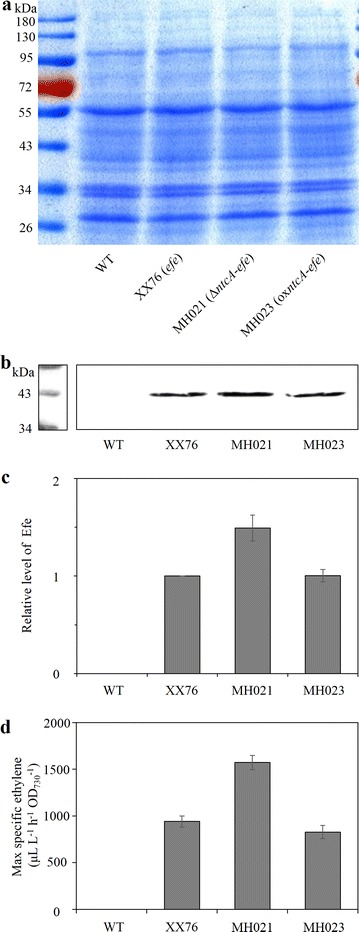
Efe level and the highest ethylene productivity. **a** SDS-PAGE showed that equal amounts of proteins were loaded in *each lane*. **b** Western blotting detection of Efe in single-copy *efe* recombinant *Synechocystis* strains. *S*. PCC 6803 WT was used as a negative control. **c** Quantitative analysis of Efe level from the results of two repeats. **d** Maximal specific productivity. Data in **d** represent the means ± standard deviations from three independent experiments

To explore why the inactivation of *ntcA* positively affected ethylene production, we first detected the level of Efe in recombinant *Synechocystis*. From western blotting and subsequent quantitative analysis, we found that strain MH021 (*ntcA* deletion in single-copy *efe*) produced 1.5-fold more Efe protein than the starting strain, XX76 (single-copy *efe*) (Fig. 4a–c). This alteration was in accordance with the 1.7-fold increase in peak specific ethylene production of MH021 (Fig. 4d). In the *ntcA* overexpression producer MH023, both the Efe level and the peak specific ethylene production were similar to those of XX76.

As the *efe* gene was under the control of P_*cpcB*_ in this study, the increased level of Efe indicated a possible direct or indirect transcriptional regulation of NtcA on P_*cpcB*_. Hence, we set up a sequence analysis of P_*cpcB*_ and found that P_*cpcB*_ might harbor a putative NtcA binding site (Additional file 1: Figure S4; Additional file 2: Table S1). The 34-bp region containing the putative NtcA binding site is directly upstream of the P_*cpc560*_ identified by Zhou et al. [32]. In addition, a broader investigation of P_*cpcB*_ from other cyanobacterial species indicated a widespread existence of putative NtcA recognition sites (Additional file 2: Table S1). Furthermore, we measured the PC content and PC/Chl a ratio of the *Synechocystis* strains with whole-cell absorbance spectra. MH015 (*ntcA* deletion) and MH021 (*ntcA* deletion in single-copy *efe*) showed increases at most of the investigated culture stages, while MH017 (*ntcA* overexpression) and MH023 (*ntcA* overexpression in single-copy *efe*) showed decreases in PC content and PC/Chl a ratio relative to *S*. PCC 6803 and XX76, respectively (Additional file 1: Figure S5a, b). Thus, improved cellular Efe levels and ethylene production are partially related to the release of negative regulation of NtcA on the P_*cpcB*_-*efe* cassette.

### NtcA modification mobilized cellular glycogen for ethylene production

**Fig. 5:**
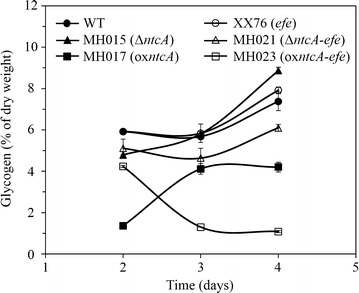
Glycogen levels of *S*. PCC 6803 WT, MH015 (*ntcA* deletion), MH017 (*ntcA* overexpression), XX76 (single-copy *efe*), MH021 (*ntcA* deletion in single-copy *efe*), and MH023 (*ntcA* overexpression in single-copy *efe*). Data represent the means ± standard deviations from three independent experiments

Since NtcA was found to regulate sugar metabolism, the glycogen content of *S*. PCC 6803 and the recombinant strains were determined to explore the relationship between cellular glycogen and ethylene production. We found that the glycogen content of *S*. PCC 6803 ranged from 6 to 7.4% of the DCW in the period corresponding to robust ethylene synthesis in the recombinant strains (Fig. 5). Compared to *S*. PCC 6803, the single-copy *efe* strain XX76 showed a similar pattern of glycogen accumulation. The deletion of *ntcA* (MH015) caused a 20% loss of glycogen on the 2nd day but rapid glycogen accumulation relative to *S*. PCC 6803, and 20% more glycogen content was observed on the 4th day (Fig. 5). As expected, MH021 (*ntcA* deletion in single-copy *efe*) showed a 14–23% reduction in glycogen content compared to XX76 (Fig. 5). In terms of the overexpression of *ntcA*, both MH017 (*ntcA* overexpression) and MH023 (*ntcA* overexpression in single-copy *efe*) showed significantly lower glycogen contents relative to their parent strains, *S*. PCC 6803 and XX76, respectively. Interestingly, the glycogen content of MH023 was much higher than that of MH017 by the 2nd day, and the glycogen accumulation trends in MH017 and MH023 were reversed; the glycogen level in MH017 was lower at the beginning and increased later, while the glycogen level in MH023 was higher at the beginning and decreased later (Fig. 5). The results of glycogen content analysis revealed that the introduction of the Efe pathway did not change the cellular glycogen level. However, the glycogen level was changed by simultaneously introducing the Efe pathway and modifying NtcA. NtcA deficiency might mobilize cellular glycogen for ethylene synthesis considering the enhanced ethylene production in MH021.

### NtcA inactivation affected the intracellular pool of succinate and 2-OG

**Fig. 6:**
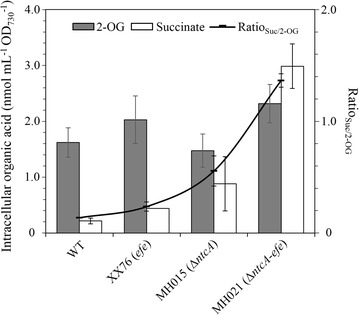
Intracellular levels of succinate and 2-OG in *S*. PCC 6803 WT, MH015 (*ntcA* deletion), MH017 (*ntcA* overexpression), XX76 (single-copy *efe*), MH021 (*ntcA* deletion in single-copy *efe*), and MH023 (*ntcA* overexpression in single-copy *efe*). Data represent the means ± standard deviations from at least two independent experiments

Succinate and 2-OG, two intermediates of the TCA cycle, are directly related to the Efe reaction. To investigate the metabolic effect of *ntcA* deletion on the unusual TCA cycle, the intracellular levels of succinate and 2-OG in the *Synechocystis* strains were monitored on the 4th day. We found that the pool of 2-OG (1.62 ± 0.26 nmol mL^−1^ OD_730_^−1^) was over 6 times larger than that of succinate (0.22 ± 0.05 nmol mL^−1^ OD_730_^−1^) under standard culture conditions in *S*. PCC 6803 WT (Fig. 6). These results confirmed the reported metabolic gap between these two metabolites, even with functional OGDC and GABA shunts [11]. In ethylene producer XX76 (single-copy *efe*), the levels of succinate and 2-OG both increased, and the ratio of succinate to 2-OG (Ratio_Suc/2-OG_) increased by 73% compared to that of *S*. PCC 6803 (Fig. 6), indicating enhanced TCA cycling, as previously noted [8]. In MH015 (*ntcA* deletion), the level of intracellular 2-OG decreased while succinate increased, and the Ratio_Suc/2-OG_ increased threefold compared to that of *S*. PCC 6803. Deleting *ntcA* in XX76 (MH021) caused a dramatic enlargement of the intracellular pool of succinate and a slight increase in 2-OG, with the Ratio_Suc/2-OG_ being enlarged by 9-, 4.8-, and 1.5-fold compared to that of *S*. PCC 6803, XX76, and MH015, respectively. These results indicated that the flux from 2-OG to succinate dramatically increased by the introduction of Efe when coupled with the partial deletion of *ntcA* and that this genetic modification reprogrammed the metabolic network of the TCA cycle.

### NtcA inactivation in combination with TCA cycle modification enhanced cellular ethylene production in recombinant *Synechocystis*

In our previous study, we constructed *Synechocystis* recombinant strain XX109 with three *efe* copies. In this recombinant, the *ogdc* (*sll1981*) and *ssadh* (*slr0370*) genes were inactivated by inserting the Km^r^-P_*cpcB*_-*efe* and Cm^r^-P_*cpcB*_-*efe* cassettes, respectively [5]. Theoretically, this strain should get an Efe-dominated TCA cycle with primarily cyclic architecture because the OGDC and GABA pathways were both blocked.

**Fig. 7:**
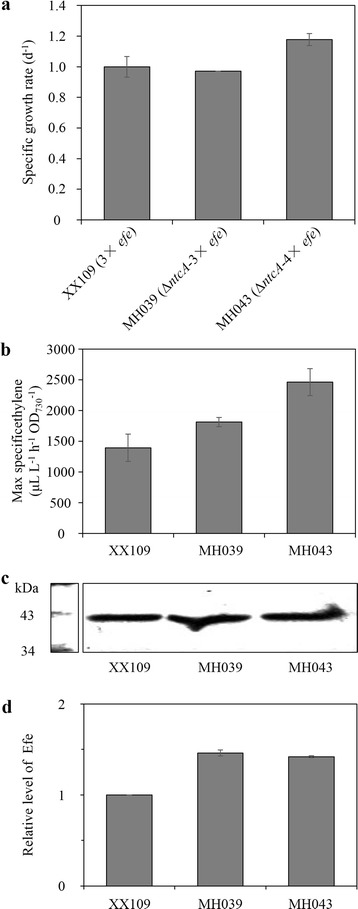
Effect of the deletion of *ntcA* on ethylene productivity and Efe level in multi-copy *efe* recombinant *Synechocystis* strains (XX109, MH039, and MH043). **a** Specific growth rate. **b** Maximal specific productivity. **c** Western blotting detection of Efe. **d** Quantitative analysis of Efe level from the results of two repeats. Data in **a** and **b** represent the means ± standard deviations from three independent experiments

To further improve ethylene production, we constructed strains MH039 and MH043 by replacing *ntcA* with a gentamycin cassette (Gm^r^) or a Gm^r^-P_*cpcB*_-*efe* cassette, respectively, based on the three-copy *efe* strain XX109 (Additional file 1: Figure S3c, d). Quantitative PCR analysis revealed that MH039 (*ntcA* deletion in three-copy *efe*) lost approximately 56% and that MH043 (*ntcA* deletion in four-copy *efe*) lost 30% of the *nctA* WT copies (Additional file 1: Figure S6). Compared to XX109, MH039 showed a similar specific growth rate, and MH043 showed a slightly improved specific growth rate (Fig. 7a). In addition, specific ethylene production was enhanced in these strains (Fig. 7b). A maximal specific ethylene production rate of 2463 ± 219 μL L^−1^ h^−1^ OD_730_^−1^ was achieved in MH043 (Fig. 7b), which was higher than the previously reported rate.

The Efe level (Fig. 7c, d) and PC content (PC/Chl a ratio as well) (Additional file 1: Figure S5c, d) both increased in MH039 (*ntcA* deletion in three-copy *efe*) and MH043 (*ntcA* deletion in four-copy *efe*) compared to that in XX109. The results confirmed the possible negative regulation of NtcA on the P_*cpcB*_-*efe* cassette, even when the OGDC and GABA shunts of the TCA cycle were blocked. However, despite harboring four *efe* copies, MH043 exhibited a similar Efe level to that of MH039.

## Discussion

Here, we enhanced ethylene production by the partial deletion of *ntcA* and analyzed the corresponding physiological and metabolic effects in *Synechocystis* strains. NtcA was the target for genetic modification based on its essential role in integrating the signals of nitrogen and carbon balance (Fig. 1). We found that *ntcA* deletion and overexpression mutants both showed a stable genotype (Fig. 2; Additional file 1: Figures S3, S6). Compared to previous studies, the lower segregation level of *ntcA* observed here might be due to different nitrogen resources. Furthermore, cell growth of the non-segregated *ntcA* mutant was not seriously restrained. Recently, it has been found that ethylene can modulate several important aspects of *Synechocystis* biology by the functional ethylene receptor (SynEtr1, encoded by *slr1212*) [33]. NtcA has also been shown to control transcription at a genome-wide level and widely affect the primary metabolism of *S.* PCC 6803. Consequently, the recovered growth of MH021 (*ntcA* deletion in single-copy *efe*) and MH023 (*ntcA* overexpression in single-copy *efe*) might relate to the combined effect of ethylene modulation and *ntcA* modification. On the other hand, the stable genotype, in combination with the recovered physiological feature, demonstrated the feasibility of improving ethylene production in *S.* PCC 6803.

Previous studies have revealed that the modification of transcription regulators can cause alterations in primary metabolism and can enhance the production of native biological products in *S*. PCC 6803 [25, 34, 35]. Thus, it is reasonable that ethylene production was affected by the deletion or overexpression of transcription factor NtcA. We first suspected that the enhanced ethylene production in MH021 (*ntcA* deletion in single-copy *efe*) (Fig. 3c, d) was related to only the cellular metabolic reprogramming imposed by *ntcA* deletion. However, Efe level detection (Fig. 4 and 7) and PC content analysis (Additional file 1: Figure S5) implied the possible native regulation of NtcA on P_*cpcB*_. Moreover, we found a putative NtcA binding site between 560 bp and 594 bp upstream of the initiation codon of the *cpcB* gene (Additional file 1: Figure S4; Additional file 2: Table S1). In fact, it has been reported that the transcription factor binding sites (TFBSs) on P_*cpcB*_ are crucial for its promoter strength in *S.* PCC 6803; however, the NtcA site was not included (only 560 bp upstream of the initiation codon was analyzed) [32]. NtcA belongs to the CRP (cAMP receptor protein) family and binds as a homodimer to the conserved palindromic sequence GTAN_8_TAC [20, 21]. As NtcA promoters were thought to serve as regulatory points to coordinate photosynthesis and nitrogen uptake [21], the genes involved in various stages of photosynthesis (such as allophycocyanin *apc*, PC *cpc*) were supposed to be new members of the NtcA regulons [21]. The possible regulation of NtcA on the P_*cpcB*_-*efe* cassette found in this study is a good example of this hypothesis.

Glycogen is the main carbohydrate storage compound that can divert a significant portion of fixed carbon in *S*. PCC 6803 [36, 37]. A rapid accumulation of glycogen usually occurs when cyanobacteria face stress conditions [37]. In this study, we observed rapid glycogen accumulation in the *ntcA* mutant MH015, but a lower level of glycogen (maximal loss of 23%) and a slow glycogen accumulation rate were observed in MH021 (*ntcA* deletion in single-copy *efe*) under standard culture conditions (Fig. 5). Thus, it is plausible that the Efe reaction can draw many carbons from glycogen when the *ntcA* transcription factor is deleted. Lower glycogen content results from enhanced catabolism and/or attenuated anabolism. It was reported that the key genes involved in glycogen catabolic pathways, such as glycolysis and the oxidative pentose phosphate (OPP) pathway, were regulated by several transcription regulators and sigma factors, including NtcA [22], Rre37 [25, 35, 38], and SigE [25, 34, 35]. The coding genes of such regulators (including NtcA itself) were among the regulons of NtcA [21, 39, 40]. Thus, under the regulation of NtcA, the intermediates involved in glycogen catabolic pathways can be converted to acetyl-CoA and malate, which are inputted into the TCA cycle. In agreement, a sharply increased Ratio_Suc/2-OG_ was found in MH021 (Fig. 6), which indicated a remodeled metabolic network for the TCA cycle. The results were also supported by identification of the predominantly cyclic architecture of the TCA cycle in recombinant *Synechocystis* with an efficient Efe pathway. In addition, the positive regulation of NtcA on the GS-GOGAT cycle of nitrogen metabolism might also bring carbon flux to the Efe reaction.

As previously found, ethylene production in cyanobacteria is a complex process involving multiple genetic and environmental factors [4–8]. The highest specific ethylene production in the multi-copy *efe* strain MH043 indicated robust metabolism in the host cells with blockage of both the OGDC and GABA pathways. Furthermore, carbon flux redistribution probably occurred, considering the alterations in glycogen content and organic acid pools. The four-copy *efe* strain MH043 showed a similar Efe level to that of MH039, which might suggest the upper limit of Efe protein expression in both strains.

## Conclusions

Based on the plasticity of the cyanobacterial metabolic network and inspired by the notion of gTME, the global nitrogen control transcription factor NtcA was chosen as the target for genetic modification to enhance ethylene production. We found that *ntcA* deficiency enhanced ethylene production in recombinant *Synechocystis*, resulting in reduced glycogen content and a drastic increase in the intracellular level of succinate. P_*cpcB*_ might be a new member of the NtcA regulons, and it exhibited a promising application prospect as a super-strong and tunable promoter in cyanobacterial biotechnology. In combination with the deletion of *ntcA* and the blockage of TCA cycle bypasses, a peak specific ethylene production rate of 2463 ± 219 μL L^−1^ h^−1^ OD_730_^−1^ was achieved in multi-copy *efe* recombinants, which is a significant increase in ethylene productivity by photoautotrophic microorganisms. The strains developed in this study and the gTME strategy can be further exploited to construct highly efficient photoautotrophic biocatalysts for ethylene production.

## Additional files

**Additional file 1: Figure S1.** Ethylene calibration curve. **Figure S2.** Schematic representation of the construction of MH013 (*ntcA* insertional inactivation). Partial coding region of *ntcA* on the genome of *S*. PCC 6803 was replaced by a kanamycin resistance (Km^r^) cassette through homologous recombination with plasmid pHM002. DNA fragments amplified by PCR and analyzed by agarose gel electrophoresis showing the partial segregation of *ntcA* in the mutant strain. PCR products from WT and mutants *S*. PCC 6803 were generated using the indicated primer pairs. Primer sequences are listed in Table 1. Lanes were loaded with PCR products that were generated with genomic DNA from the indicated strains as template. The sizes of the PCR products are indicated on the right. **Figure S3.** Construction of MH021 (*ntcA* deletion in single-copy *efe*), MH023 (*ntcA* overexpression in single-copy *efe*), MH039 (*ntcA* deletion in three-copy *efe*) and MH043 (*ntcA* deletion in four-copy *efe*). (a) and (c) Schematic representation. (b) and (d) Genotypic confirmation by PCR and agarose gel electrophoresis. PCR products from WT and mutants *S*. PCC 6803 were generated using the indicated primer pairs. Primer sequences are listed in Table 1. Lanes were loaded with PCR products that were generated with genomic DNA from the indicated strains as templates. The DNA marker with relevant sizes (in bp) was indicated on the left. **Figure S4.** Schematic representation of putative NtcA site on P_*cpcB*_ of *S*. PCC 6803. **Figure S5.** Effect of *ntcA* deletion or overexpression on pigment contents. (a) and (c) Absorbance spectra for the 2nd, 3rd and 4th day. Peaks of PC and Chl a are indicated. Groups of spectra were shifted along the y-axis for better viewing. (b) and (d) PC/Chl a ratios as a function of time. XX76 (single-copy *efe*), MH015 (*ntcA* deletion), MH017 (*ntcA* overexpression), MH021 (*ntcA* deletion in single-copy *efe*), MH023 (*ntcA* overexpression in single-copy *efe*), XX109 (three-copy *efe*), MH039 (*ntcA* deletion in three-copy *efe*), MH043 (*ntcA* deletion in four-copy *efe*). **Figure S6.** Quantitative PCR results of *ntcA* deletion in MH039 (*ntcA* deletion in three-copy *efe*) and MH043 (*ntcA* deletion in four-copy *efe*). The copy numbers of *ntcA* were measured through qPCR. The reference genes for *S*. PCC 6803 were *rnpB* and the 16S rRNA gene. The relative ratio of gene copy numbers of *ntcA* were quantified in XX109 (three-copy *efe*), MH039 and MH043. Data represent the means ± standard deviations from at least two independent experiments.

**Additional file 2: Table S1.** Putative NtcA binding sites upstream of the *cpcB* gene from different cyanobacteria.

## Abbreviations

Efe: ethylene-forming enzyme
gTME: global transcription machinery engineering
2-OG: 2-Oxoglutarate
TCA cycle: tricarboxylic acid cycle
OGDC: 2-Oxoglutarate decarboxylase
GABA: γ-Aminobutyrate
PHB: polyhydroxybutyrate
GS: glutamine synthetase
GOGAT: glutamine oxoglutarate aminotransferase
WT: wild type
ORF: open reading frame
PCR: polymerase chain reaction
qPCR: quantitative real-time PCR
PC: phycocyanin
Chl a: chlorophyll a
DCW: dry cell weight
OD: optical density
Ratio_Suc/2-OG_: ratio of succinate to 2-OG
TFBSs: transcription factor binding sites
RP: cAMP receptor protein
OPP: oxidative pentose phosphate pathway
