# Supplementary material for: Effects of global transcription factor NtcA on photosynthetic production of ethylene in recombinant Synechocystis sp. PCC 6803
Source: Biotechnol Biofuels. 2017 Jun 6;10:145. doi: 10.1186/s13068-017-0832-y (PMC5460508; doi:10.1186/s13068-017-0832-y)
Supplement: Supplementary file 1 — Additional file 1: Figure S1. Ethylene calibration curve. Figure S2. Schematic representation of the construction of MH013 (ntcA insertional inactivation). Partial coding region of ntcA on the genome of S. PCC 6803 was replaced by a kanamycin resistance (Kmr) cassette through homologous recombination with plasmid pHM002. DNA fragments amplified by PCR and analyzed by agarose gel electrophoresis showing the partial segregation of ntcA in the mutant strain. PCR products from WT and mutants S. PCC 6803 were generated using the indicated primer pairs. Primer sequences are listed in Table 1. Lanes were loaded with PCR products that were generated with genomic DNA from the indicated strains as template. The sizes of the PCR products are indicated on the right. Figure S3. Construction of MH021 (ntcA deletion in single-copy efe), MH023 (ntcA overexpression in single-copy efe), MH039 (ntcA deletion in three-copy efe) and MH043 (ntcA deletion in four-copy efe). (a) and (c) Schematic representation. (b) and (d) Genotypic confirmation by PCR and agarose gel electrophoresis. PCR products from WT and mutants S. PCC 6803 were generated using the indicated primer pairs. Primer sequences are listed in Table 1. Lanes were loaded with PCR products that were generated with genomic DNA from the indicated strains as templates. The DNA marker with relevant sizes (in bp) was indicated on the left. Figure S4. Schematic representation of putative NtcA site on PcpcB of S. PCC 6803. Figure S5. Effect of ntcA deletion or overexpression on pigment contents. (a) and (c) Absorbance spectra for the 2nd, 3rd and 4th day. Peaks of PC and Chl a are indicated. Groups of spectra were shifted along the y-axis for better viewing. (b) and (d) PC/Chl a ratios as a function of time. XX76 (single-copy efe), MH015 (ntcA deletion), MH017 (ntcA overexpression), MH021 (ntcA deletion in single-copy efe), MH023 (ntcA overexpression in single-copy efe), XX109 (three-copy efe), MH039 (ntcA deletion in three-copy efe) [file 13068_2017_832_MOESM1_ESM.docx]

**Figure S1** Ethylene calibration curve.

**Figure S2** Schematic representation of the construction of MH013 (*ntcA* insertional inactivation). Partial coding region of *ntcA* on the genome of *S*. PCC 6803 was replaced by a kanamycin resistance (Km^r^) cassette through homologous recombination with plasmid pHM002. DNA fragments amplified by PCR and analyzed by agarose gel electrophoresis showing the partial segregation of *ntcA* in the mutant strain. PCR products from WT and mutants *S*. PCC 6803 were generated using the indicated primer pairs. Primer sequences are listed in Table 1. Lanes were loaded with PCR products that were generated with genomic DNA from the indicated strains as template. The sizes of the PCR products are indicated on the right.

**Figure S3** Construction of MH021 (*ntcA*deletion in single-copy *efe*), MH023 (*ntcA* overexpression in single-copy *efe*), MH039 (*ntcA*deletion in three-copy *efe*) and MH043 (*ntcA*deletion in four-copy *efe*). (a) and (c) Schematic representation. (b) and (d) Genotypic confirmation by PCR and agarose gel electrophoresis. PCR products from WT and mutants *S*. PCC 6803 were generated using the indicated primer pairs. Primer sequences are listed in Table 1. Lanes were loaded with PCR products that were generated with genomic DNA from the indicated strains as templates. The DNA marker with relevant sizes (in bp) was indicated on the left.

**Figure S4** Schematic representation of putative NtcA site on P*_cpcB_* of *S*. PCC 6803.

**Figure S5** Effect of *ntcA* deletion or overexpression on pigment contents. (a) and (c) Absorbance spectra for the 2nd, 3rd and 4th day. Peaks of PC and Chl a are indicated. Groups of spectra were shifted along the y-axis for better viewing. (b) and (d) PC/Chl a ratios as a function of time. XX76 (single-copy *efe*), MH015 (*ntcA* deletion), MH017 (*ntcA* overexpression), MH021 (*ntcA*deletion in single-copy *efe*), MH023 (*ntcA* overexpression in single-copy *efe*), XX109 (three-copy *efe*), MH039 (*ntcA*deletion in three-copy *efe*), MH043 (*ntcA*deletion in four-copy *efe*).

**Figure S6** Quantitative PCR results of *ntcA* deletion in MH039 (*ntcA*deletion in three-copy *efe*) and MH043 (*ntcA*deletion in four-copy *efe*). The copy numbers of *ntcA* were measured through qPCR. The reference genes for *S*. PCC 6803 were *rnpB* and the 16S rRNA gene. The relative ratio of gene copy numbers of *ntcA* were quantified in XX109 (three-copy *efe*), MH039 and MH043. Data represent the means ± standard deviations from at least two independent experiments.
