# Supplementary material for: Effects of global transcription factor NtcA on photosynthetic production of ethylene in recombinant Synechocystis sp. PCC 6803
Source: Biotechnol Biofuels. 2017 Jun 6;10:145. doi: 10.1186/s13068-017-0832-y (PMC5460508; doi:10.1186/s13068-017-0832-y)
Supplement: Supplementary file 2 — Additional file 2: Table S1. Putative NtcA binding sites upstream of the cpcB gene from different cyanobacteria. [file 13068_2017_832_MOESM2_ESM.docx]

**Additional file 2:**

**Table S1.** Putative NtcA binding sites upstream of the *cpcB* gene from different cyanobacteria

| **Genome** | **ORF** | **Putative NtcA motif**  (**GTA**N_8_**TAC**N_22-23_**TA**N_3_**T**/**A**) | **NtcA site position**^a^ |
| --- | --- | --- | --- |
| *Synechococcus elongatus* PCC 7942 | *Synpcc7942_1052* | **GTA**tcagagat**GAC**N_22_**TA**ctc**T** | -327 |
| *Synechococcus elongatus* PCC 6301 | *syc0496*_*c* | **GTA**tcagagat**GAC**N_22_**TA**ctc**T** | -327 |
| *Anabaena* sp. strain 90 | *ANA*_*C11068* | **GTA**taaacata**AAC**N_22_**AA**agt**T** | -242 |
| *Synechococcus* sp. PCC 7002 | *SYNPCC7002*_*A2209* | **GTA**taacattt**ACC**N_22_**TA**ctc**C** | -338 |
| *Leptolyngbya* sp. O-77 | *O77CONTIG1*_*03742* | **GTT**tatccgcg**TAC**N_22_**TA**gct**G** | -132 |
| *Synechocystis* sp. PCC 6803 | *sll1577* | **GTT**ataaaata**AAC**N_22_**TA**gag**A** | -591 |
| *Nostoc* sp. PCC 7120 | *alr0528* | **GTT**ttaattca**GAC**N_22_**GA**tta**A** | -47 |
| *Anabaena variabilis* ATCC 29413 | *Ava*_*2930* | **GTT**aaacgaca**GAC**N_22_**TT**cac**A** | -230 |
| *Synechococcus* sp. JA-3-3Ab | *CYA*_*2043* | **GGA**gcttccac**TTC**N_22_**TA**acc**T** | -71 |
| *Thermosynechococcus elongatus* BP-1 | *tlr1957* | **GCT**taatttat**CTC**N_22_**TA**act**A** | -345 |
| *Thermosynechococcus* sp. NK55 | *NK55*_*06480* | **GAC**ctttccgt**CAC**N_22_**TA**aaa**A** | -194 |
| *Acaryochloris marina* MBIC11017 | *AM1*_*C0098* | **GAC**tgaattag**ACC**N_22_**TA**cat**T** | -386 |

^a^start from the first codon
